# Integrating artificial intelligence into African health systems and emergency response: Need for an ethical framework and guidelines

**DOI:** 10.4102/jphia.v16i1.876

**Published:** 2025-03-31

**Authors:** Nicaise Ndembi, Bethanie Rammer, Joseph Fokam, Uttam Dinodia, Sofonias K. Tessema, Jean Philbert Nsengimana, Sarah Mwangi, Edem Adzogenu, Landry Tsague Dongmo, Bleddyn Rees, Brian O’Connor, Trevor A. Crowell, Wilmot James, Vittorio Colizzi, Ngashi Ngongo, Jean Kaseya

**Affiliations:** 1 Africa Centres for Disease Control and Prevention (Africa CDC), Addis Ababa, Ethiopia; 2 African Society for Laboratory Medicine (ASLM), Addis Ababa, Ethiopia; 3 Avacare Global Health, Lusaka, Zambia; 4 Faculty of Medicine and Biomedical Sciences, University of Yaounde I, Yaounde, Cameroon; 5 United Nations Children’s Fund (UNICEF), Addis Ababa, Ethiopia; 6 European Connected Health Alliance, Dublin, Ireland; 7 Henry M. Jackson Foundation for the Advancement of Military Medicine, Bethesda, Maryland, United States of America; 8 Brown University School of Public Health, Department of Health Services, Policy and Practice, Providence, Rhode Island, United States of America; 9 UNESCO Board of Biotechnology and Bioethics, University of Rome Tor Vergata, Rome, Italy

## Artificial intelligence in the African health sector

Artificial intelligence (AI) is defined as computer systems capable of performing tasks normally associated with human cognition. Artificial intelligence tools have expanded into various disciplines over the last decade, including genomics, proteomics, metabolomics, epidemic preparedness and response, biosecurity and weather forecasting.^1^ The current generation of AI technologies can generate human language with remarkable fluency and make robust predictions. In the health sector, such developments allow both big data synthesis and analyses to inform individual patient care and population-level policies. Furthermore, AI tools have the potential to facilitate patient and provider engagement, simplify healthcare administration and improve efficiency through process automation.^2,3^ Artificial intelligence thus offers a myriad of opportunities for making healthcare accessible, cost-effective, innovative and better at improving results for communities across the globe, including in low- and middle-income countries (LMICs) such as those found in Africa.

Approximately 80% of adults in Africa own a mobile phone, 33% of which are smartphones, and they are comfortable using mobile phones for a wide variety of tasks.^4^ This is an asset for widespread adoption of AI in Africa at the consumer level. Although the adoption of AI into global health sectors is still in its preliminary stages, there are some notable examples of its successful implementation, at scale, across African health sectors in Kenya, Morocco, Nigeria, Ghana, Ethiopia and South Africa.^5,6,7,8,9^ The Africa Centres for Disease Control and Prevention (CDC) Pathogen Genomics Initiative has also generated a large amount of data that can be used for modelling and outbreak attribution. Greater AI integration into African healthcare will likely aid coordinated responses to public health threats. According to the Africa Centres for Disease Control and Prevention (Africa CDC), every year Africa experiences over 160 public health emergencies, most of which are infectious diseases,^10^ and 75% of emerging infectious diseases are zoonotic.^11^ Artificial intelligence could significantly accelerate the promise that the world can prepare for such threats, with major improvements for pathogen detection and outbreak attribution, strengthening global early warning systems, accelerating vaccine design, optimising clinical trials and development, enhancing biomanufacturing processes and attributing outbreak origins.

In this way, AI could constitute a useful addition to more traditional detection methods. Specifically, AI could contribute to early disease recognition and characterisation – critical for rapid design of future-proofed countermeasures – as well as Infection Prevention and Control (IPC). Once an outbreak has been recognised, AI methods could help identify genetic variants, annotate genes, predict both structure and function of proteins and detect anomalies in sequencing data, thus assisting with determination of likely sources of the outbreaks and tracing routes of emergence and transmission. Given the frequency of emerging and re-emerging zoonotic events, the early disease recognition and characterisation that AI tools can facilitate could prove invaluable, enabling rapid design of future-proofed countermeasures and facilitating infection prevention. In fact, AI metapopulation models were used to implement the ‘Partnership for Evidence-Based Response to COVID-19 (PERC)’ study and inform coronavirus disease 2019 (COVID-19) response efforts on the African continent.^12^ The PERC assessed the effectiveness and impact of COVID-19 public health measures by integrating epidemiological and social measures data from several African Union Member States^12^ to analyse social behavioural parameters associated with COVID-19 at the neighbourhood level for 20 selected cities of interest. These data included the position of a device, such as a mobile phone, in latitude and/or longitude coordinates and the time the device was at that location. In this case, AI was able to show that mobility was the key to a quantitative understanding of the emergence of waves and thus helped to target interventions with limited resources.

## Challenges for artificial intelligence integration into health systems

Artificial intelligence could revolutionise healthcare in Africa; however, thorough and careful design, development and oversight are essential for AI to improve health outcomes across the continent. Efforts towards this include one of the flagship objectives of Africa CDCs digital transformation strategy, ‘AI for Health in Africa’, which is supported by a wide range of industry, government, experts and development partner organisations. The Digital Health Society’s AI Club, a group with members from pharmaceutical and technology companies, regulators, academics, healthcare consultants and policymakers, published a report in September 2023 with six concise recommendations that resonate strongly in the African context.^13^ These recommendations urge the creation of global frameworks to safeguard various critical aspects of AI including evidence-based monitoring and evaluation; evaluation of risks and opportunities related to sensitive topics such as demographics; health priorities and ethics; the skills and education required for the healthcare workforce to competently use AI; the health literacy required by civil society; and standards for transparency and explainability in AI health implementations.^13^ The African Union High-level Emerging Technologies Committee launched a white paper, the Continental Artificial Intelligence Strategy, a strategic blueprint and roadmap of the African Union’s AI strategy for the continent, as a first step towards such a framework for Africa.^14^

Africa faces various challenges in harnessing AI-based technologies, including high costs associated with computing infrastructure installation, insufficient data on health and diseases in African populations and underrepresentation in global clinical research. Such disparities undermine the global health community’s ability to develop effective interventions across diverse populations. Low bandwidth internet remains a major barrier to the effective uses of cloud-based computing solutions in Africa. The average broadband internet speed in Africa was 21.12 Mbps in 2023, which is far below the global average of 72.7 Mbps. Additional challenges include lack of stakeholder trust in AI, limited specialist AI knowledge and/or expertise and a lack of governance and/or guidance for AI integration or for both individual and population-level data usage and security.^15,16,17,18,19^ In Africa, the aforementioned challenges are further complicated by the continent’s multifaceted ethical, legal and social realities, including widely varied demographics and infrastructure (Table 1). A comprehensive Africa CDC-initiated framework must, therefore, cover product life cycles, ethics and data governance for the development of a sophisticated, ethical and culturally sensitive AI system for African health applications (Table 2). The roadmap must also include plans for the scaled-up development of specialised AI-trained workforces, through improved and wide-ranging science, technology, engineering and mathematics (STEM) education that incorporates AI in the curriculum of public health and medical schools, and continuous skills development following qualification. This is a particular concern as the Government AI Readiness Index 2021 ranked Africa as one of the regions with the lowest level readiness for AI adoption, with an overall score of 3.49 out of 10, ranking fifth out of the six regions assessed.^20^

**TABLE 1:** Challenges to the development, deployment and integration of artificial intelligence in Africa.

Challenge	Considerations
Technical challenges	Diverse infrastructure/Interoperability Data network limitations Insufficient workforce skillsets Widespread lack of access to computing resources Data quality Cybersecurity
Ethical challenges	Informed consent Safety Privacy standards and regulations Dataset and algorithm oversight Equity
Legal challenges	Liability Misuse Privacy regulations Data protection regulations Accountability frameworks for professional negligence and computational errors
Social challenges	Trust building Cultural sensitivity and inclusion Alignment with societal norms
Inclusivity	Demographics Economic status Equity

**TABLE 2:** Framework for development and integration of artificial intelligence into African health systems.

Action		Description
1.	Needs assessment and goal definition	Identify challenges in Africa and set precise objectives for AI systems in healthcare.
2.	Stakeholder engagement and collaboration	Engage diverse stakeholders, including software developers, clinicians, administrators, medical researchers, data scientists, policy makers, private sector entities, patients, healthcare practitioners and community members, to tailor AI systems to African healthcare needs.
3.	Data collection and analysis	Collect, curate, document and analyse high-quality, diverse data to minimise inherent biases and ensure the integrity and generalisability of AI applications generated from these data.
4.	Technology selection and development	Choose appropriate AI technologies considering the African healthcare context, scalability and the goals of the technology.
5.	Testing and validation	Establish AI system efficacy through real-world testing and validation using data that are relevant to the African healthcare setting in which the technology will be deployed.
6.	Regulatory compliance and approval	Ensure compliance with standards and obtain necessary approvals. Establish regulatory mechanisms.
7.	Implementation and training	Educate healthcare professionals and other potential users on AI system operation and integration.
8.	Continuous monitoring and improvement	Monitor AI systems for sustained efficacy, safety and relevance by systematically analysing quantitative performance data and undertaking qualitative assessments, checking for anticipated outcomes and digging for insights from any unanticipated outcomes.
9.	Documentation of experiences	Document deviations from pre-specified plans and other experience with deployment of the AI system to improve understanding of real-world performance and enhance future AI development and deployment efforts.

AI, artificial intelligence.

## Important considerations for artificial intelligence integration into health systems in Africa

Artificial intelligence poses a significant risk of grave harm if harnessed and misused deliberately or accidentally across sectors. Within Africa and globally, a common position on health data governance is required with the aim of embracing a ‘single digital health market’ that accelerates AI applications for broad societal benefit while simultaneously preventing misuse and reducing risks.

The algorithms used by AI tools can only be as precise as the quality and diversity of the underlying population data, including cultural, gender, ethnicity, age and the digital infrastructure used to train them. Africa’s demographics are incredibly varied, which means that if multiple representative groups are not included in the development of AI tools, then the resulting solutions will not be deployable across the continent. Thus, African health systems seeking to integrate AI tools will require diverse local and regional data for training, cautious application deployment and rigorous performance evaluation. Usability and cultural adaptation are facilitated via transparency; therefore, benchmarking must be established and recorded for quality and openness.

Another important consideration for AI deployment in Africa is the need to ensure data safety and privacy through appropriate regulatory frameworks. Policymakers will need to build the legal instruments that enable digital innovations to thrive while continuously balancing protection of the population against potential risks of the technology’s misuse. Some algorithms have been trained on massive datasets to interpret and respond via text or language outputs. These ‘large language models’ (LLMs) can understand text prompts given by a user and provide a response. Some LLMs are considered ‘generative AI’, meaning they can also generate complex text outputs in response to a prompt, drawing from information available on the Internet and other inputs, as seen with programmes such as ChatGPT. These represent powerful tools for synthesising and summarising diverse and highly technical information.^21^ However, they are also vulnerable to misuse. Anti-vaccine campaigns proliferated during the COVID-19 pandemic, and vaccine hesitancy driven by misinformation and disinformation on social media are one example. After implementation, AI technologies will need regulatory and technical frameworks to ensure patient and child safety and system stability through regular maintenance and monitoring of system resilience. A thorough cybersecurity-focussed risk management approach is crucial to maintain the robustness of these systems over time. Safe and ethical use of AI in healthcare requires integration and proactive risk management, especially cybersecurity. The risk management of Artificial Intelligence in healthcare must span the whole product lifecycle. This strategy must adapt to cybersecurity threats. Health data are precious; thus, AI systems must utilise innovative security, vulnerability evaluations and data privacy rules. An effective risk management strategy is crucial.

Finally, African AI systems must be verified against global reference standards to ensure healthcare quality, patient outcomes and resource allocation. To create a responsible AI system for healthcare, version control and audit trails must be confidential. Software developers, clinicians, healthcare administrators, health researchers, data scientists, policymakers, patients and civil society organisations must work together to create AI solutions that build on equity principles and meet African healthcare objectives.

## Conclusion

The African continent is particularly well-positioned to adopt AI applications and harness their benefits in the workforce development and health security space due to their critical role in advancing preparedness infrastructure on the continent and enabling the rapid growth of capability across both healthcare and health security. Implementing these recommendations will allow Africa to contribute to, and benefit from, AI’s ethical, equitable, and responsible development.

The views and opinions expressed in this article are those of the author’s (L.T.N) and do not necessarily reflect the official policy or position of UNICEF.
